# A bibliometric analysis of the study of urban green spaces and health behaviors

**DOI:** 10.3389/fpubh.2022.1005647

**Published:** 2022-09-26

**Authors:** Sining Zhang, Xiaopeng Li, Zhanglei Chen, Yu Ouyang

**Affiliations:** ^1^Department of Landscape Architecture, School of Architecture, Southwest Jiaotong University, Chengdu, China; ^2^School of Architecture and Urban Planning, Chongqing University, Chongqing, China

**Keywords:** Citespace, knowledge mapping, bibliometric analysis, urban green spaces, health behaviors

## Abstract

Urban green space can supply a range of ecosystem services and general health benefits for people. This paper reviewed and analyzed 607 papers related to urban green space and health behaviors from 2002 to 2021 in the Web of Science core collection by using Citespace 6.1.R2 software. The scientifically bibliometric analysis and visual analysis were conducted to analyze the basic characteristics, literature co-citation analysis, research hotspots, and frontier trends. The findings show that 11 co-citation clusters indicate the research intellectual base. Also, 19 main keywords with a high frequency and 20 main keywords with a high centrality were extracted. Burst detection analysis reveals three research frontier trends: the correlation between urban green space and health behavior; the driving and impact factors; and the study of environmental justice and social equity. This paper aims to systematically review the progress and basic situation of urban green spaces and health behaviors research around the world, which helps to gain a comprehensive understanding of this field, as well as provide value and references for subsequent research.

## Introduction

Globally, there is no such universal definition of urban green space. Its definition varies according to context or geographic range. Generally, urban green space (UGS) includes parks, gardens, street greenery, wetlands, community green spaces, natural woodland, etc., which is recognized as the optimized mechanism for human living environments and life quality (1, 2). UGS can deliver varieties of ecosystem services and general health benefits for people, especially for women and the old (3, 4), by encouraging various physical activity behaviors and nature contact (5–8).

World Health Organization (9) (WHO) defined “health” as “a state of complete physical, mental and social wellbeing, not merely the absence of disease or infirmity”. Currently, the increasing frequency of global public health events, such as COVID-19 (confirmed 504.4 million by April 20, 2022), and the huge burden of disease, such as there were 13.1% age-standardized prevalence of obesity among adults in 2016 (10), has gained more international attention and academic concern. A number of studies have explored and explained the relationship between UGS and human health behavior (HB), concerning physical and mental health. Most studies proved that UGS has a positive influence on HB. For example, a previous WHO report states that UGS can positively affect physical activity and mental health (11). More specifically, greater use and coverage of UGS in residential communities can improve behavioral development, reduce rate of Attention of Deficit Hyperactivity Disorder in children (12), help restorative psychological effects (13), and reduce the feelings of loneliness (14). Also, streetscape greenery positively affects old adults' walking propensity and travel ability (15–17). Urban wildscapes are positively associated with adults' perceived restoration, stress, and mental health (18). Besides, the complex UGS system (i.e., the horizontal, vertical greenery, and proximity of green levels) could reduce residents' obesity (19).

Yet, existing reviews mainly focus on the factors that affect the relationship between UGS and HB, such as the exposure to UGS air pollution and health (20), certain ecosystem services and health (21), UGS and walking (22, 23). The literature on integrating research of UGS and HB in the review article group remains limited. A scientifically bibliometric analysis of the general UGS and HB research is required. In this paper, the publications related to UGS and HB from 2002 to 2021 were analyzed by using Citespace 6.1.R2 software, while contributing to the ongoing discussion. The remainder of this paper is organized as follows. Section Materials and methods presents the methodology of this study. Section Results provides a coherent knowledge base regarding the basic characteristics (distribution of years, journals, disciplines, areas, countries, and institutions), literature co-citation analysis, research hotspots, and frontier trends combined with the visual knowledge maps. In conclusion, implications and limitations, and future development prospects are discussed in section Discussion. The main findings in this paper were concluded in section Conclusion. This study aims to systematically review the progress and basic situation of UGS and HB research around the world, in order to provide a scientific reference for the related research.

## Materials and methods

### Data collection

A preliminary search in the Web of Science Core Collection (WoSCC) database was conducted to select the keywords. Then, the retrieval type was: (TS = “urban green space” AND “health behavior”) OR (TS = “urban park” AND “health behavior”) OR (TS = “urban green infrastructure” AND “health behavior”) OR (TS = “urban green” AND “health behavior”), only concerning the original research article and review. Timespan (almost 20 years): 2002-01-01−2021-12-31 (since there is no product in 2001), language = English. Search time was July 19, 2022. Finally, 607 publications were obtained, including 553 articles and 54 review articles, according to relevance and then removed the duplications.

### Methods

**Figure 1:**
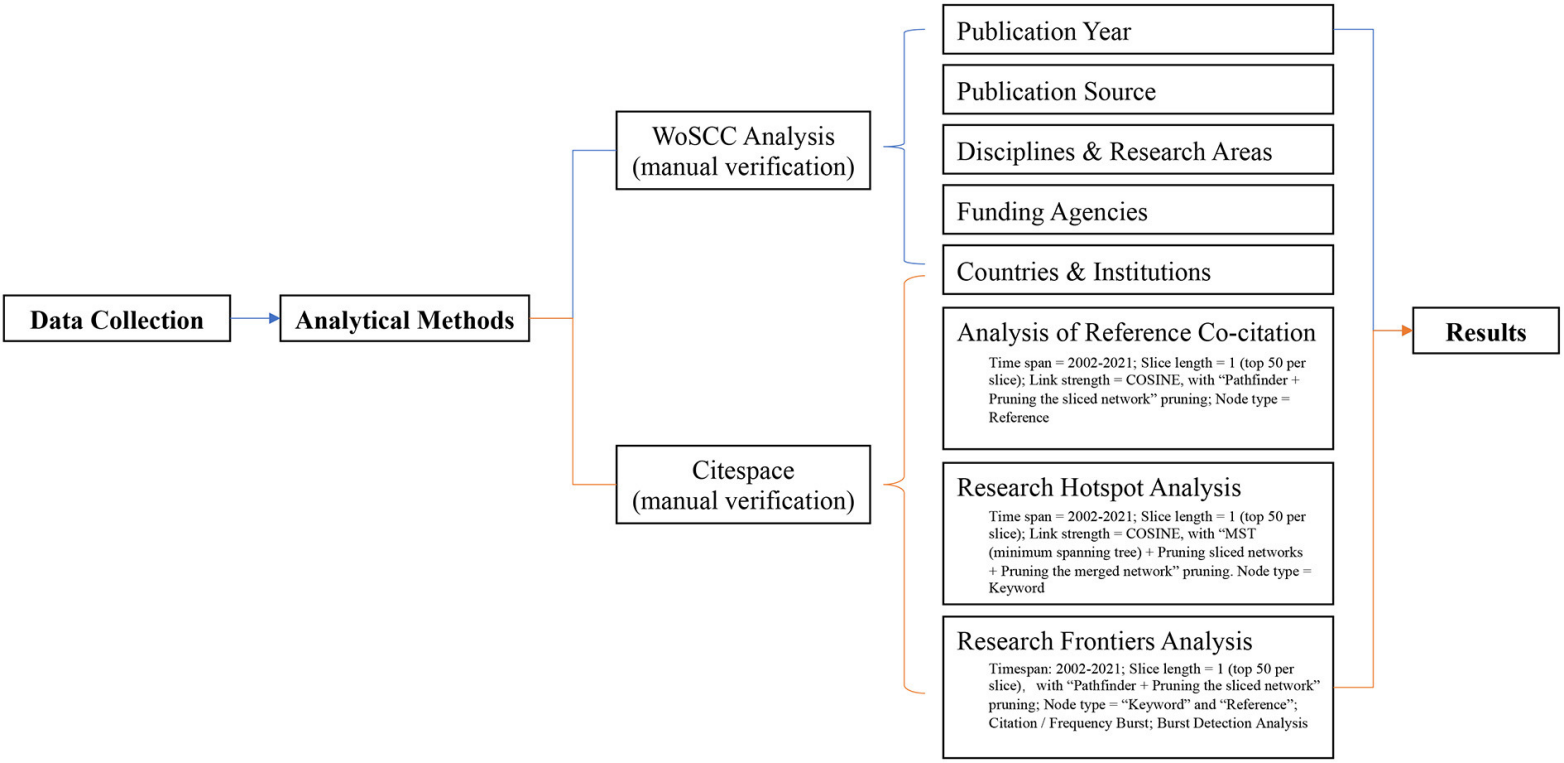
Flow chart of analytical methods.

Citespace (Citation Space) is a software that combines scientometric analysis and visual analysis, which can present the structure, rules, and distribution of scientific knowledge. Also known as the “Mapping Knowledge Domains, MKD” (24, 25). Six hundred and seven references were imported into Citespace 6.1.R2, and then were further analyzed regarding the publication output and journal distribution, the development of disciplines and research areas, distribution by countries and institutions, co-citation analysis, research hotspots, and frontiers analysis, by setting and modulating the relevant parameters (see Figure 1). Figure 1 shows that the process of research methodology in this paper.

## Results

### Profile of publication

#### Publication output and journal distribution

**Figure 2:**
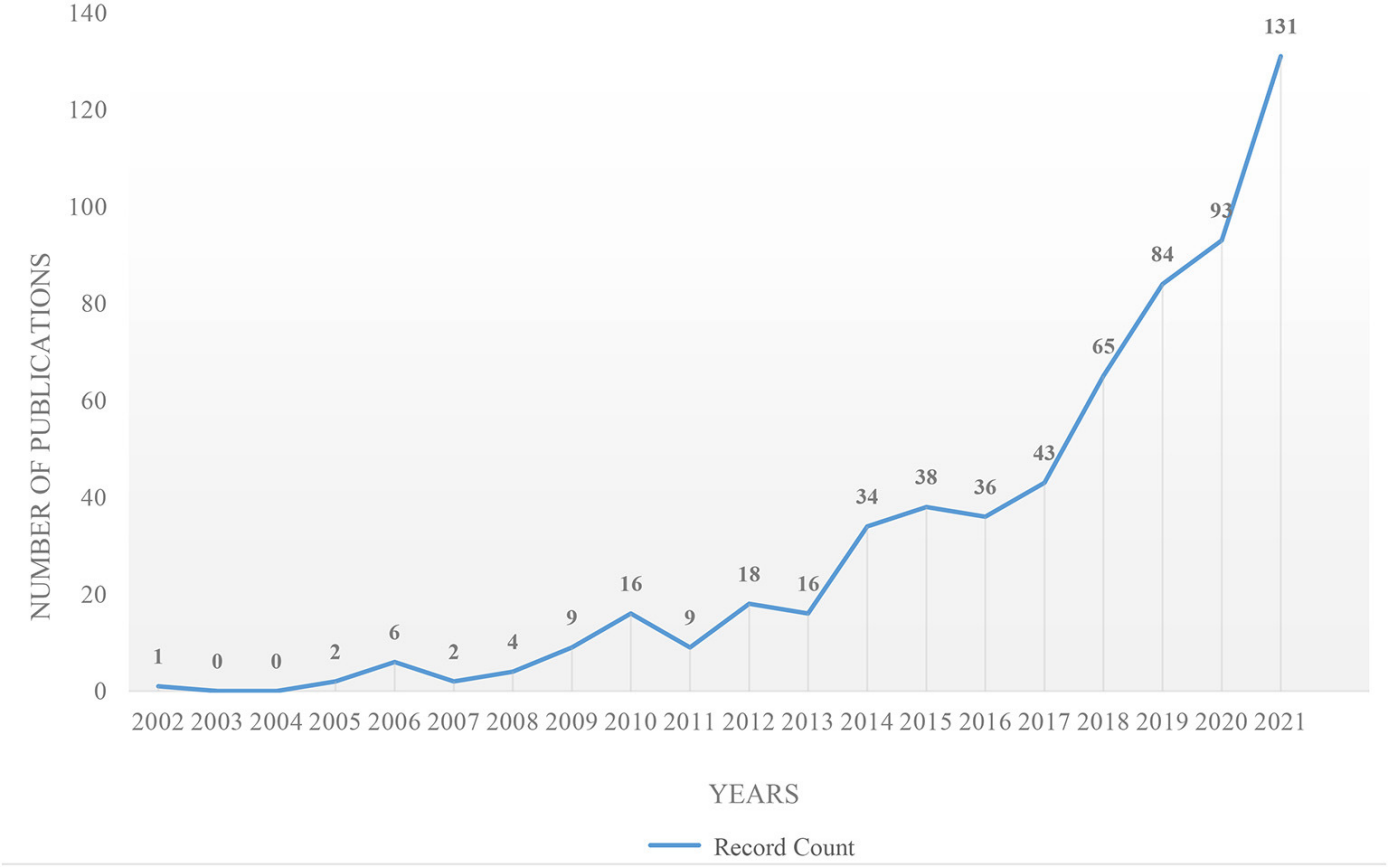
Literature distribution by years concerning the study of urban green space and health behaviors. Graphical representation based on WOS Core Collection search on July 19th, 2022.

As can be seen from Figure 2, literature publication generally shows a growing trend from 2002 to 2021, consisting of two periods: a slow growth period and a rapid growth period. First, from 2002 to 2013, it showed a slow growth trend, during which there were three times of decline (2006–2007; 2010–2011; and 2012–2013). During this period, only 83 articles were published, accounting for 13.67%. But in the period of rapid growth (from 2014 to 2021), there were 524 publications, increasing by as much as 8-fold compared to the first period. This may be related to people's increasingly strong pursuit of health awareness and outcomes.

Regarding the study of UGS and HB, the papers were mainly published in journals concerning public, environmental & occupational health, environmental research, and urban studies. Table 1 shows the main productive source publications. *International Journal of Environmental Research and Public Health, Urban Forestry Urban Greening*, and *Landscape and Urban Planning* are the top three productive journals. Among these, *Science of the Total Environment* has the highest impact factor (10.753). Articles were published in these ten journals that accounted for about 32% of the total.

**Table 1:** Top 10 productive source publications.

**Source titles**	**Impact factor (JCR 2021)**	**Record count**	**% Of 607 publications**
*International Journal of Environmental Research and Public Health*	4.614	51	8.402
*Urban Forestry Urban Greening*	5.766	35	5.766
*Landscape and Urban Planning*	8.119	21	3.460
*Sustainability*	3.889	18	2.965
*Health & Place*	4.931	16	2.636
*International Journal of Behavioral Nutrition and Physical Activity*	8.915	15	2.471
*Journal of Physical Activity & Health*	3	12	1.977
*PLoS ONE*	3.752	12	1.977
*Science of the Total Environment*	10.753	12	1.977
*BMC Public Health*	4.135	11	1.812

JCR, journal citation reports.

#### Development of disciplines and research areas

Although the amount of literature obtained from the retrieval results is small, the studies involve 93 disciplines in total. There are six categories with articles over 40: Public Environmental Occupational Health (194), Environmental Sciences (148), Environmental Studies (116), Urban Studies (82), Green Sustainable Science Technology (43), and Geography (40), respectively. Moreover, the research field involves 73 research areas, of which, the area of Environmental Sciences Ecology accounts for the highest occupation (247 articles).

#### Distribution by countries and institutions

**Figure 3:**
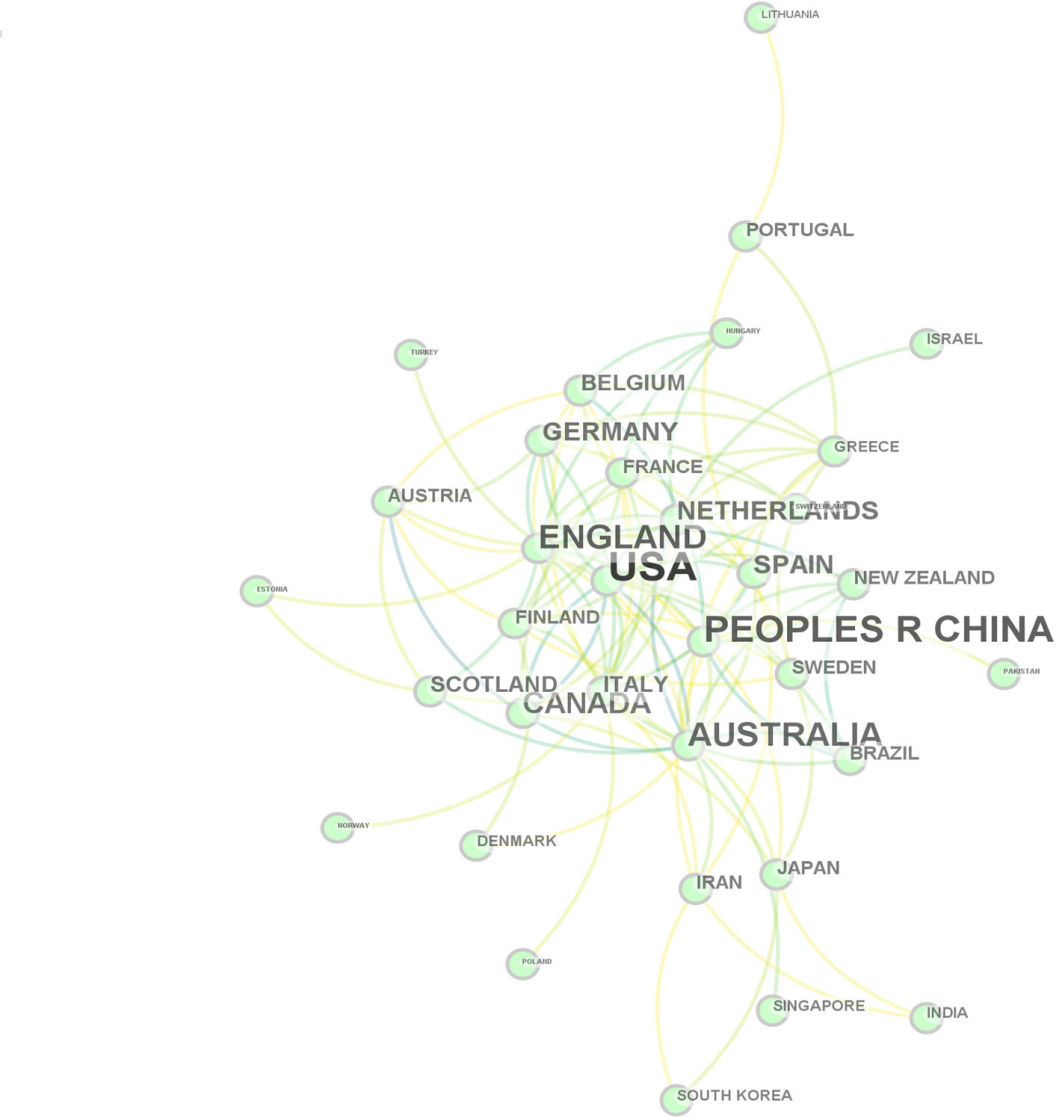
Countries of origin of articles.

The publications are from 74 countries and regions in the world. Table 2 presents the top ten most productive countries. Although the United States had the highest number of publications (193), it ranked fifth (0.18) in terms of betweenness centrality value, as the same as China (0.18). Australia ranked fourth regarding the record count (66). Its betweenness centrality value was the highest (0.37), indicating that Australia is in the most critical position in the cooperative network. Italy (0.34), England (0.22) and the Netherlands (0.21) followed. Also, from Figure 3, we can see that European countries form the most important network of cooperative relationships. In addition, China is the only Asian country in the top 10 countries/regions. “the National Natural Science Foundation Of China” ranked fourth among the top scientific research funding institutions. This may be related to the fact that the Communist Party officially put forward the “consciousness of advocating a community with a shared future for mankind” at its 18th National Congress in 2012. The mainstreaming of this consciousness has led scholars to pay more attention to human health.

**Table 2:** The top ten productive countries or regions.

**Countries/regions**	**Record count**	**% Of 607**	**Centrality**
USA	193	31.796	0.18
Peoples R China	98	16.145	0.18
England	91	14.992	0.22
Australia	66	10.873	0.37
Canada	38	6.260	0.04
Netherlands	35	5.766	0.21
Germany	34	5.601	0.04
Spain	33	5.437	0.14
Scotland	26	4.283	0.02
Italy	18	2.965	0.34

**Figure 4:**
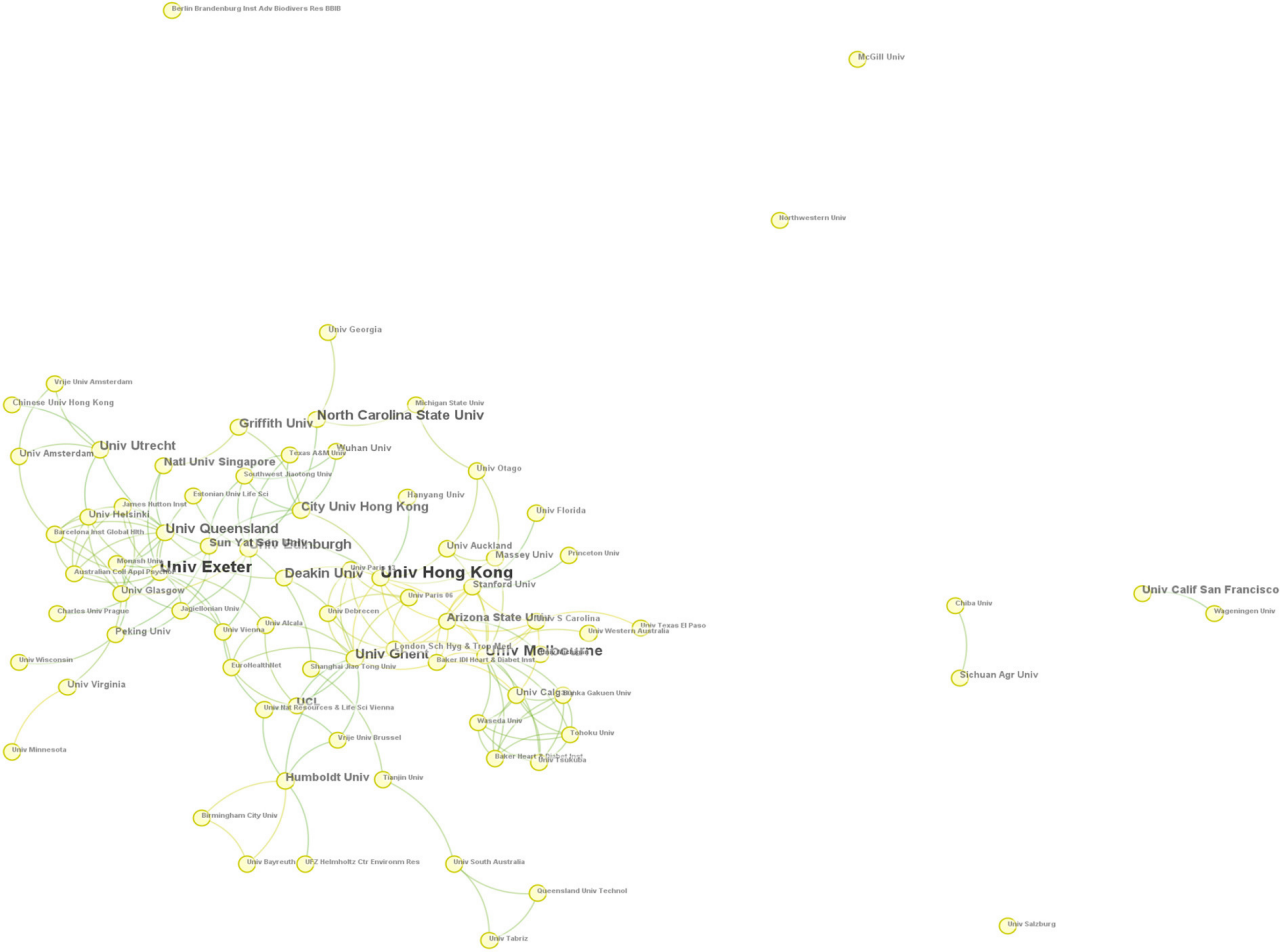
Institutions of origin of articles.

A cooperation network of institutions produced 120 nodes (Figure 4), indicating the authors are from 120 research institutions. The top three productive institutions are the University of Hong Kong (14), the University of Exeter (14), and the University of Melbourne (11). The top 50 are dominated by the United States (15), Europe (13), Asia (9), Australia (6), Canada (3), New Zealand (3), and Brazil (1). It can be seen that the related studies are almost concentrated in European countries, North America, Australia, and Asia. A few in Latin America. The results are evenly distributed in space. Figure 4 also shows that a relatively close cooperative network has been formed among various academic institutions.

### Analysis of reference co-citation

A and B are the co-cited relationship when papers A and B appear together in the reference list of a third cited paper (25). Similar studies can be aggregated by literature co-citation analysis to form major research areas, which shape the citation network that can trace the development context forward and explore the research frontier backward, as well as form the knowledge base of the specific research field. In all, 607 publications were analyzed in Citespace. Eleven clusters were extracted by using the clustering algorithm. They were labeled by the first term from the LLR (log-likelihood ratio, p-level) algorithm, numbered from #0 to #10 (Table 3). In Table 3, the silhouette value of each cluster is >0.7, indicating that the clustering reliability is very high. It is worth mentioning that the larger the silhouette value, the higher the similarity of the cluster members. Among the 11 clusters, the average year of nine clusters was 2015 or later, indicating that most of the clusters (related research) are relatively new. The mean year of publication in Cluster #6 and Cluster #9 was 2020, named “COVID-19” and “utilizing big data”, which is related to COVID-19 pandemic in 2019, and the increasing use of internet big data. Moreover, 11 clusters actually reflect the intellectual basis of the UGS and HB research. The largest top three clusters are “human health”, “park use”, and “residents' perception”, showing that the main research base is under the human health, park use, and residents' perception research fields.

**Table 3:** Summary of 11 clusters.

**ID**	**Size**	**Silhouette**	**Mean (year)**	**Top terms (log-likelihood ratio)**
#0	86	0.75	2017	**Human health** (66.83, 1.0E-4); New Zealand adolescent (63.1, 1.0E-4); objective neighborhood environment (63.1, 1.0E-4)
#1	78	0.785	2015	**Park use** (92.9, 1.0E-4); urban design (79.16, 1.0E-4); park environment (71.12, 1.0E-4)
#2	74	0.92	2011	**Residents' perception** (118.29, 1.0E-4); walkable community (118.29, 1.0E-4); adjacent park (113.72, 1.0E-4)
#3	66	0.881	2016	**Physical activity** (84.24, 1.0E-4); ecological justice perspective (62.07, 1.0E-4); broad view (62.07, 1.0E-4)
#4	55	0.884	2017	**Urban greenness** (96.55, 1.0E-4); walking behavior (91.07, 1.0E-4); urban green space (58.61, 1.0E-4)
#5	46	0.903	2019	**Edible forest garden** (79, 1.0E-4); salutogenic affordance (79, 1.0E-4); multiple benefit (79, 1.0E-4)
#6	37	0.923	2020	**COVID-19 pandemic** (341.23, 1.0E-4); vulnerable communities (79.55, 1.0E-4); outdoor recreation (74.49, 1.0E-4)
#7	29	0.895	2017	**Green open space development model** (55.04, 1.0E-4); dense urban setting (55.04, 1.0E-4); green space behavior (47.88, 1.0E-4)
#8	27	0.944	2014	**Urban neighborhood** (62.29, 1.0E-4); socioeconomic gradient (58.45, 1.0E-4); neighborhood park (58.45, 1.0E-4)
#9	23	0.999	2020	**Utilizing big data** (70.43, 1.0E-4); city center (44.62, 1.0E-4); smart cities (44.62, 1.0E-4)
#10	15	0.938	2011	**Residential neighborhood** (54.99, 1.0E-4); spatial contagion (54.99, 1.0E-4); influencing participation (45.11, 1.0E-4)

**Figure 5:**
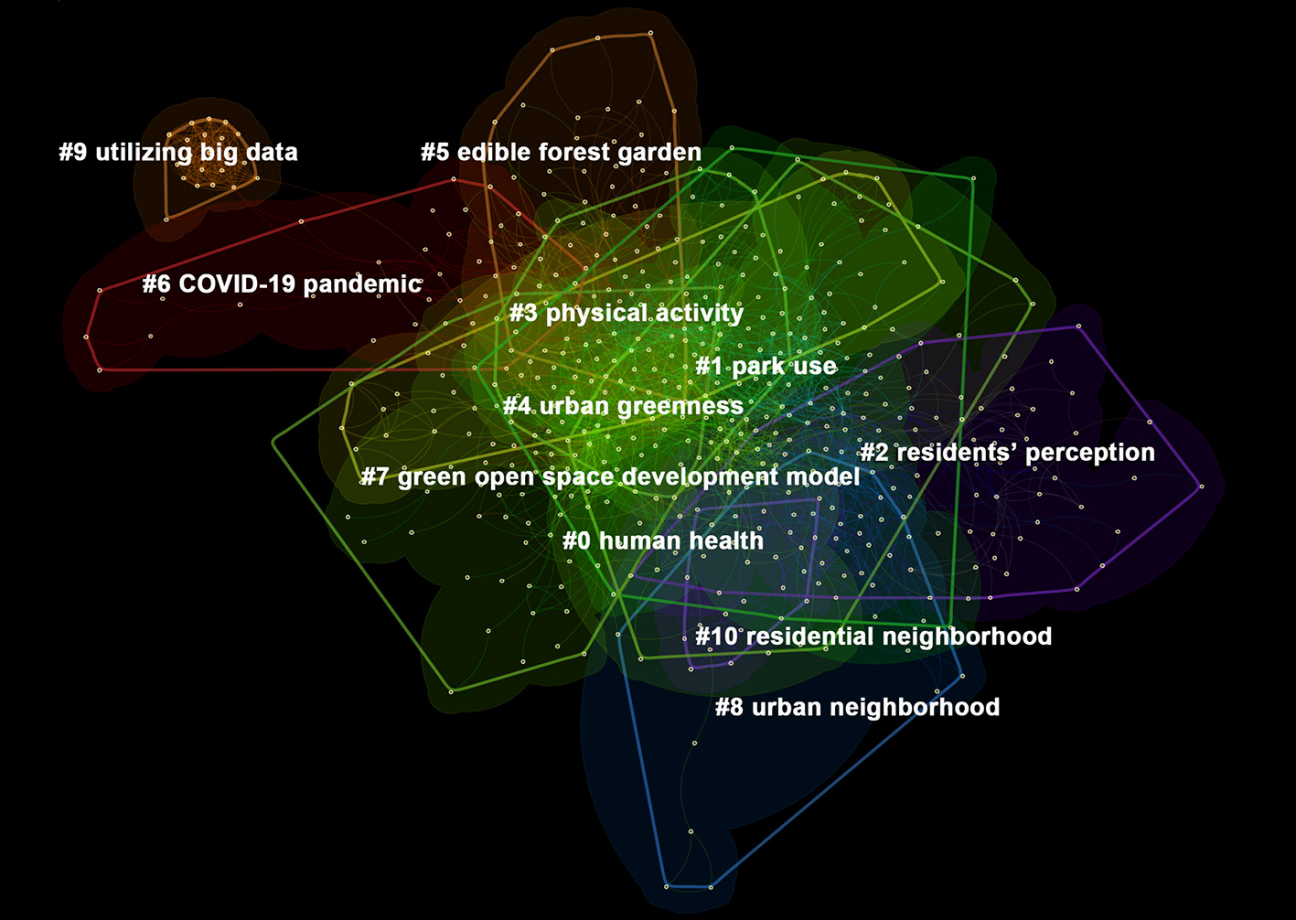
Knowledge map of co-cited reference analysis.

The co-citation analysis map was achieved (Figure 5). In Figure 5, different colors represent different clusters. Nodes represent different publications, and lines represent network relationships between the publications. Figure 5 shows the complex integration among 11 clusters, indicating that they are not independent studies under 11 research fields, but also cross-over studies.

Besides, the top ten highly-cited publications in the sample range are listed in Table 4. The paper “Nature and Health” published by Hartig et al. (13) is significant since it has the highest citation frequency (66). Besides, regarding the frequency higher than 20, there are four articles in Cluster #1, two each in Cluster #3 and Cluster #4, and one each in Cluster #0 and Cluster #2.

**Table 4:** Top ten highly-cited references.

**Frequency**	**Cluster**	**Title**	**References**
66	#3	Nature and Health	Hartig et al. (13)
45	#1	Urban green space, public health, and environmental justice: the challenge of making cities “just green enough”	Wolch et al. (5)
30	#4	Exploring pathways linking greenspace to health: theoretical and methodological guidance	Markevych et al. (26)
28	#1	The health benefits of urban green spaces: a review of the evidence	Lee and Maheswaran (27)
26	#3	More green space is linked to less stress in deprived communities: evidence from salivary cortisol patterns	Ward et al. (28)
24	#0	A Review of the Health Benefits of Greenness	James et al. (29)
23	#1	The impact of interventions to promote physical activity in urban green space: a systematic review and recommendations for future research	Hunter et al. (30)
21	#2	Effect of exposure to natural environment on health inequalities: an observational population study	Mitchell and Popham (31)
20	#1	Opportunity or Orientation? Who Uses Urban Parks and Why	Lin et al. (32)
20	#4	Streetscape greenery and health: stress, social cohesion and physical activity as mediators	De Vries et al. (33)

#### Cluster #0: Human health

Cluster #0 is the largest cluster (86 members), appearing dark green in Figure 5. Cluster #0 was formed from 2008 to 2019, and the average year of publication is 2017, which means this is a new cluster. It quickly became the largest cluster in a very short period of time. This cluster focused on human health, highlighting the key role of the urban green environment in human welfare.

There are four articles that have a citation frequency higher than 10 in this cluster. Moreover, a paper by James et al. (29), “A Review of the Health Benefits of Greenness” had the highest citation count in Cluster #0 (34). It found strong evidence for a positive correlation between greenness and physical activity. Besides, the article “Advantages of public green spaces in enhancing population health” by Sugiyama et al. (35) was the most active paper, since it cited 26 papers from Cluster #0. This paper focuses on the important relationship between public green spaces and health benefits, as well as three advantages of using public green spaces as a health promotion measure.

#### Cluster #1: Park use

Cluster #1 was labeled as “park use” with 78 members. The publication years of this cluster are from 2006 to 2019 (Mean year = 2015). The citation counts of eleven papers exceed 10. This cluster mainly focuses on the studies on how the general or the specific characteristics of parks could affect their usage for residents.

The article by Wolch et al. (5), has the highest citation count, also the third most cited article among 607 papers. It emphasizes the key role of urban green space in public health. It also explained the paradoxical nature of urban green space strategies, whereby creating new green spaces to make neighborhoods healthier (e.g., environmental justice issues), is accompanied by an increase in housing costs.

Furthermore, Sugiyama et al. (35) is still the most active literature that cited 17 papers in Cluster #1. And then the paper “The associations between park environments and park use in southern US communities” by Banda et al. (36), cited 15 papers in Cluster #1.

### Research hotspots analysis

**Figure 6:**
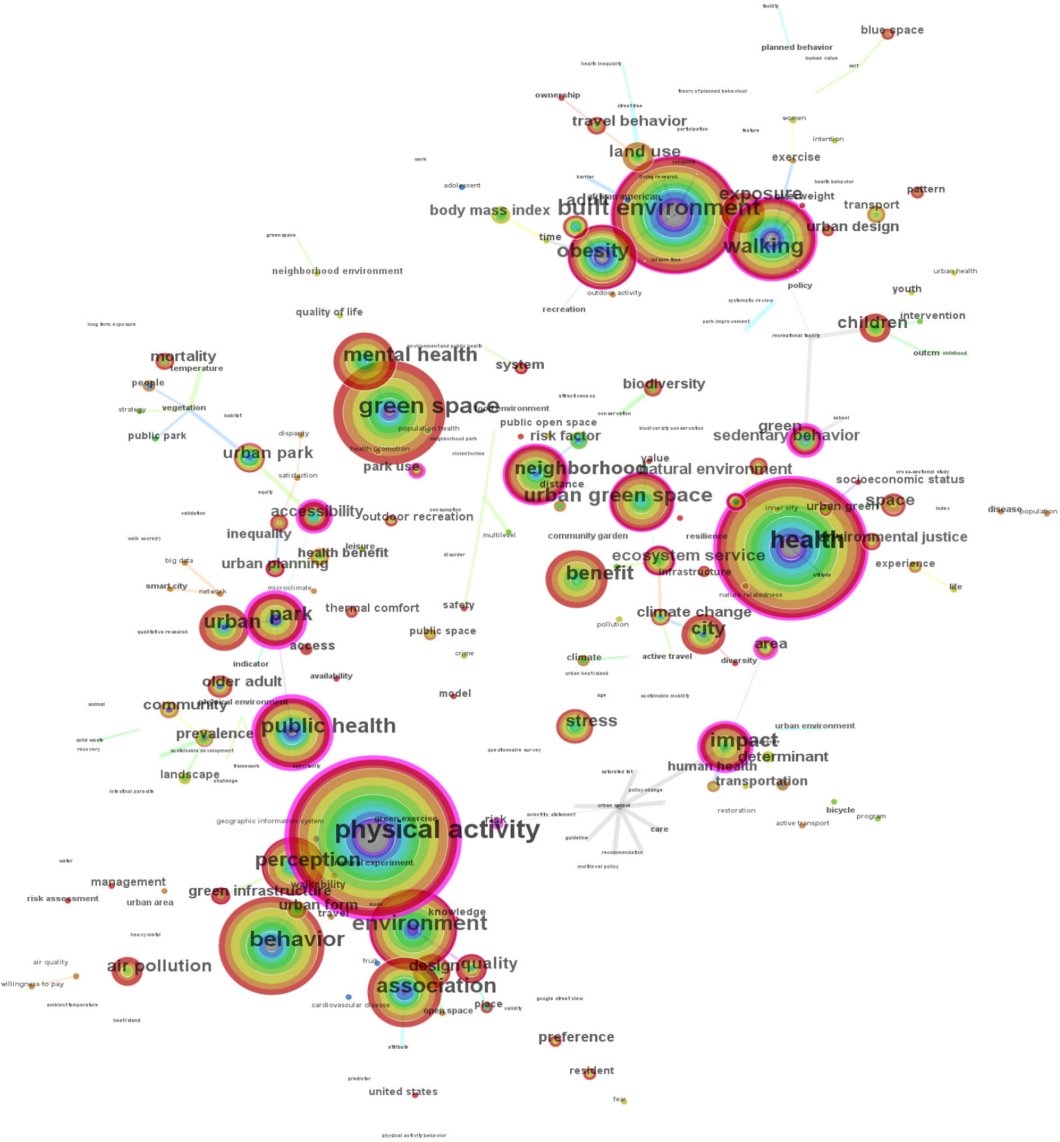
Keywords co-occurrence network.

Keywords co-occurrence analysis refers to the co-occurrence relationship between two terms when they appear jointly in one paper. The higher the frequency of co-occurrence, the stronger the relationship between the two keywords. Through the co-occurrence analysis of keywords, hot topics in a given field can be analyzed. Then, a co-occurrence analysis map of keywords was obtained (Figure 6). There are 216 nodes (i.e., 216 critical keywords), and 105 links (density = 0.0045).

In Figure 6, the circle node represents the frequency. The higher the frequency, the larger the circle. The purple nodes in the outer ring indicate the highest betweenness centrality. The largest circle node is “physical activity”, with a count of 229, as well as the highest betweenness centrality value of 1.29. It also was the first important keyword appearing in 2005. Moreover, in Table 5, 19 keywords were extracted, since they had a frequency >40, showing that research on such areas has become increasingly popular. Twenty keywords were extracted and ranked by betweenness centrality, indicating that studies on such fields have been a research focus. According to Table 5 and Figure 6, we can see that the keywords mainly involve three research objects: (1) Different scales of UGS, like urban parks, neighborhood parks, recreational facilities, and so on; (2) Different health behaviors and outcomes, such as walking, sedentary behavior, obesity, and mental health; (3) The impact factors, e.g., accessibility, impact, risk.

**Table 5:** Main keywords ranked by the frequency and the betweenness centrality value.

**Count**	**Year**	**Keyword**	**Centrality**	**Year**	**Keyword**
229	2005	Physical activity	1.29	2005	Physical activity
119	2007	Health	1.07	2009	Park
116	2010	Green space	1.05	2010	Public health
112	2008	Built environment	0.98	2011	Urban sprawl
100	2010	Behavior	0.94	2011	Impact
87	2008	Environment	0.94	2006	Risk
72	2009	Walking	0.84	2015	Accessibility
72	2010	Public health	0.82	2007	Health
70	2008	Association	0.82	2008	Area
59	2012	Urban green space	0.8	2009	Sedentary behavior
52	2009	Park	0.8	2009	Recreational facility
51	2010	Mental health	0.77	2009	Walking
51	2009	Obesity	0.72	2015	Park use
51	2011	Impact	0.63	2014	Neighborhood
50	2008	Perception	0.53	2012	Urban green space
50	2016	Benefit	0.5	2008	Built environment
47	2010	Urban	0.33	2009	Obesity
46	2014	Neighborhood	0.3	2008	Environment
44	2012	City	0.26	2016	Ecosystem service
			0.21	2016	Natural environment

### Research frontiers analysis

**Figure 7:**
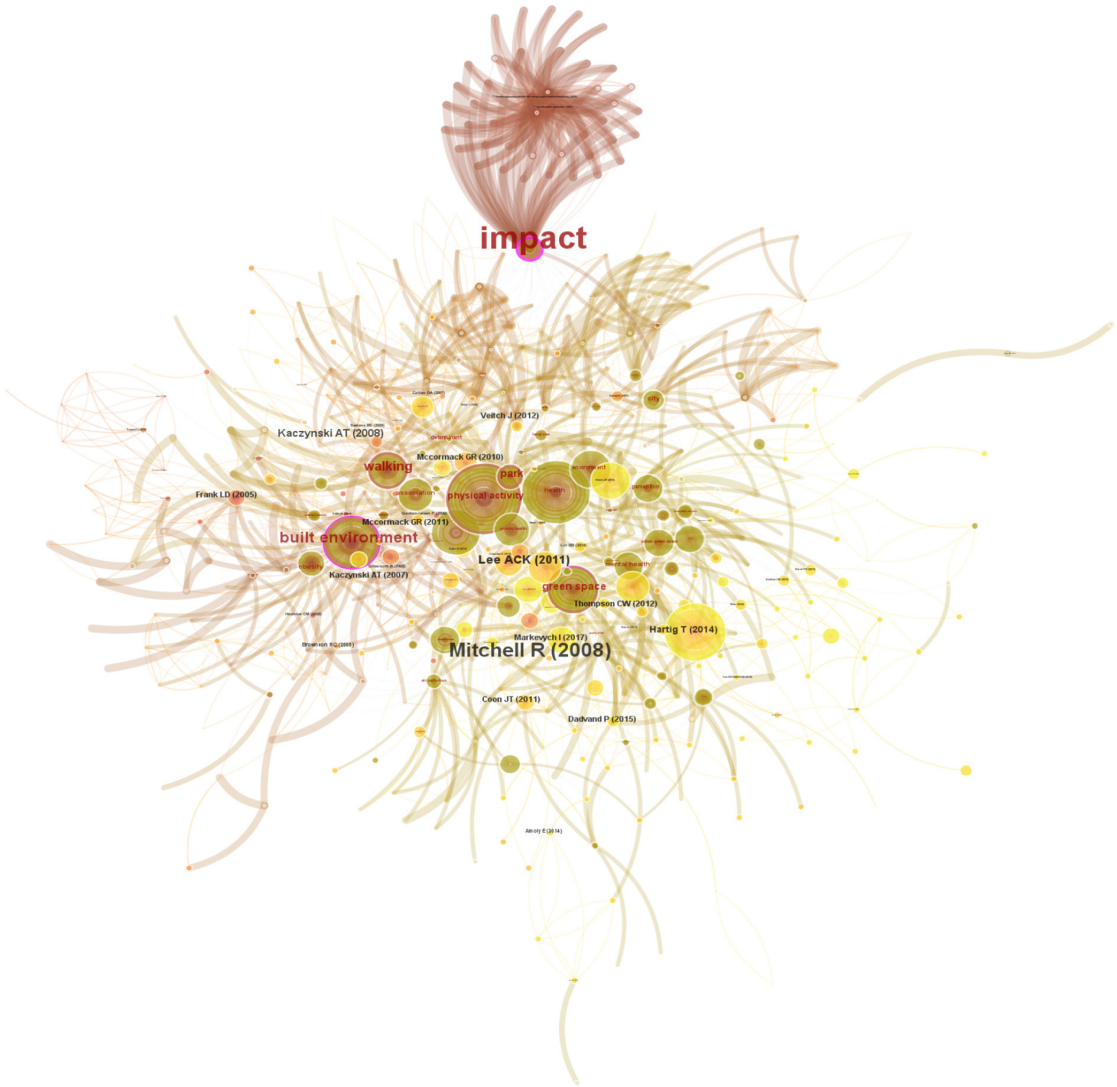
Map of burst detection of emerging terms and references.

Burst detection can capture nodes with great frequency changes in a certain period of time. Such mutation information can reflect the rise of a research theme, which is often used to do cutting-edge analysis. A map of burst detection of emerging terms and references was achieved (Figure 7). There are 504 nodes and 3,364 links (density = 0.0265). Light yellow nodes represent the reference, and dark yellow nodes represent the terms, in proportion to the degree of betweenness centrality. So, the term “built environment” and the paper by Mitchell and Popham (31) have the highest centrality value.

A total of 9 keywords were extracted (Table 6). As mentioned above, “physical activity” is the first key term that is confirmed again here. Moreover, “built environment” has the strongest strength of citation bursts (8.7), and its influence lasted from 2008 to 2013. However, no important keywords with strong citation burst were added after 2018. Furthermore, the top nine keywords with the strongest citation burst also reflect the research frontiers related to UGS and HB, including driving factors (e.g., built environment, risk factors, public parks, intervention); and the dimensions of health (e.g., physical activity, walking, obesity).

**Table 6:** Top 9 keywords with the strongest citation bursts from 2002 to 2021.

**Keywords**	**Strength**	**Begin**	**End**	**2002–2021**
Built environment	**8.7**	2008	2013	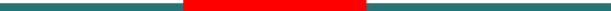
Physical activity	**5.23**	2005	2008	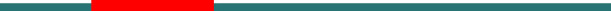
Health	**4.98**	2007	2012	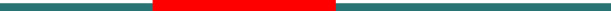
Walking	**4.94**	2009	2016	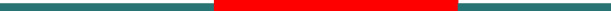
Obesity	**4.94**	2009	2014	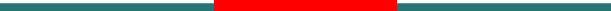
Risk	**4.34**	2006	2011	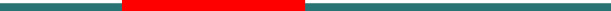
Risk factor	**4.28**	2014	2018	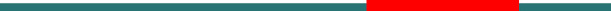
Intervention	**3.4**	2017	2018	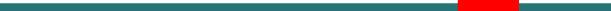
Public park	**3.24**	2014	2017	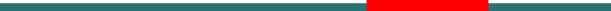

The blue line represents the time measure (2002–2021), which is made up of 19 blue line segments; The red line shows the time period of the citation bursts (from begin to end).

Moreover, Table 7 shows the top 15 references with the strongest citation bursts in the WoSCC database from 2002 to 2021. Among them, the strength of the citation bursts started from 2008 to 2021. From 2019 to 2021, there are three papers with high strength in the citation bursts: Wolch et al. (5); Frumkin et al. (37); and Baran et al. (38), indicating that they are nearly 3 years of cutting-edge research.

**Table 7:** Top 15 references with the strongest citation bursts in WoSCC database from 2002 to 2021.

**References**	**Title**	**Strength**	**Begin**	**End**	**2002–2021**
Frank et al. (39)	Linking objectively measured physical activity with objectively measured urban form: findings from SMARTRAQ	**9.13**	2009	2013	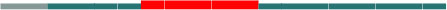
Mitchell and Popham (31)	Effect of exposure to natural environment on health inequalities: an observational population study	**8.41**	2009	2016	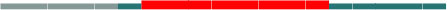
Giles-Corti et al. (40)	Increasing walking: how important is distance to, attractiveness, and size of public open space?	**6.42**	2010	2013	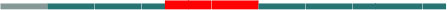
Maas et al. (41)	Green space, urbanity, and health: how strong is the relation?	**6.35**	2010	2014	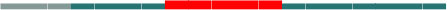
McCormack et al. (42)	Characteristics of urban parks associated with park use and physical activity: a review of qualitative research	**5.97**	2014	2017	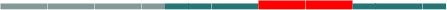
Saelens and Handy (23)	Built environment correlates of walking: a review	**5.58**	2011	2015	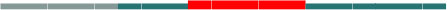
Wolch et al. (5)	Urban green space, public health, and environmental justice: the challenge of making cities “just green enough”	**5.26**	2019	2021	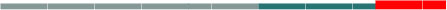
Gordon-Larsen et al. (43)	Inequality in the built environment underlies key health disparities in physical activity and obesity	**5.09**	2008	2014	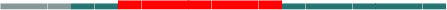
Kaczynski and Henderson (44)	Environmental correlates of physical activity: a review of evidence about parks and recreation	**4.75**	2010	2014	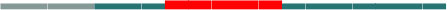
Kaczynsk et al. (45)	Association of park size, distance, and features with physical activity in neighborhood parks	**4.54**	2010	2016	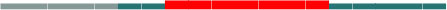
Frumkin et al. (37)	Nature Contact and Human Health: a Research Agenda	**4.51**	2019	2021	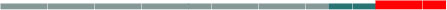
Tzoulas et al. (46)	Promoting ecosystem and human health in urban areas using Green Infrastructure: a literature review	**4.35**	2012	2015	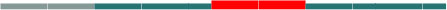
Cohen et al. (47)	Contribution of public parks to physical activity	**4.29**	2010	2015	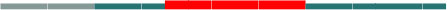
Owen et al. (22)	Understanding environmental influences on walking: review and research agenda	**4.17**	2010	2012	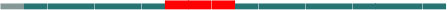
Baran et al. (38)	Park use among youth and adults: examination of individual, social, and urban form factors	**4.1**	2019	2021	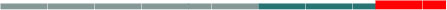

The light blue line shows the time measure; The dark blue line means the year of publication, up to 2021; The red line shows the time period of the citation bursts (from begin to end).

The top 15 articles further reveal the research frontiers, integrating with the other publications with strong citation bursts. Hence, there are three research frontiers regarding the study of UGS and HB in the nearly two decades: (1) the relationship between UGS and HB; (2) the influence factors; (3) environmental justice and social equity.

## Discussion

### Implications and limitations

The findings show that the number of publications increased from 2002 to 2021. And it has increased significantly since 2014, which is closely related to the global organizations supporting national efforts with much-sophisticated assistance. For instance, *Global Action Plan* was published in 2014 (34), and the 17 United Nations Sustainable Development Goals (SDG) were adopted in 2015. More specifically, SDG 3 “good health and well-being” advocates for all countries to guarantee and improve people's health. Also, SDG 11.7 aims to achieve the following: “By 2030, provide universal access to safe, inclusive and accessible, green and public spaces, in particular for women and children, older persons and persons with disabilities” (48). Furthermore, the most important jump happens from 2019 to 2021, which is a period facing a global public health crisis COVID-19, that may indicate a motivation for academia to focus on health behaviors research.

Besides, most published journals belong to the medical category (e.g., “public, environmental & occupational health”, “medicine”, “general & internal”) and the environmental sciences and ecology category (e.g., “environmental sciences”, “urban studies”). Herewith, the natural sciences are dominant in the research of UGS and HB. There are few studies related to social science. A discipline that intersects the natural and social sciences is involved, such as psychology. So, in future research, some sociocultural aspects of UGS, such as aesthetics and historical culture, should be considered for their impact on human health behavior.

Eleven clusters extracted in this paper provide a knowledge base for the research areas of UGS and HB. Through in-depth analysis, some articles are highly cited (Table 4), which is also confirmed by the burst detection analysis (Figure 7). Hence, as a knowledge base, the highly cited articles in the clusters still have enough influence on the current research.

From the keyword co-occurrence analysis in Section Research hotspots analysis, three research objects demonstrate that the keywords mainly focus on the UGS types and scales, HB types and outcomes, and the influencing factors. Yet, there are few keywords related to different human groups (e.g., age, gender, socioeconomic status, race), landscape and ecological characteristics (e.g., aesthetic value, cultural services, biodiversity), and social issues (e.g., landscape equity and justice).

Although considerable research supports that UGS has a positive influence on health, the theoretical and conceptual frameworks for how UGS provides benefits to human health and wellbeing are limited (49). Besides, at present, urban green space design for human health is mostly concentrated in the field of medical and rehabilitation design, which ignores the continuity and correlation of different UGS. Many findings could provide references for urban spatial planning, landscape design, and decision-making (49, 50). Hence, the co-work of landscape architects, urban planners, and designers is essential to devote themselves and their professional knowledge and experience to the maximized and optimized planning and design of UGS concerning the health benefits, combined with scientific studies.

Yet, this paper has some limitations. First, this study only collected publications in the WoSCC database, rather than using multiple databases to collect a larger sample of studies. Also, we only select English articles for analysis. Therefore, some non-English papers will be ignored. Second, due to space limitations, some literature clusters and nodes need to be further analyzed in detail.

### Future research trend

First, Plenty of studies have been conducted to explain the relationship between green space and human wellbeing. From the perspective of positive correlation, green space is beneficial to improving people's health and wellbeing (46, 47), both in physical health and mental health. For instance, the percentage of green space in a resident's living environment is positively correlated with their perceived health (41). Besides, Sugiyama et al. (51) evidenced that perceived community greenness was more strongly associated with mental health than physical health, by using a sample of 1,895 adults with physical and mental health scores in the Adelaide of Australia. Although many studies proved that nature contact and UGS may offer a range of human health benefits (13, 37, 44), there is still much evidence that remains unknown. Conversely, green space also leads to negative outcomes, such as the potential pathogenic effects of UGS. Hence, the relationship between UGS and HB is complex and interactive, which is one of the future research trends.

Second, there is comparatively little evidence proving that specific health benefits are associated with certain features of UGS, though in the huge body of literature in this research area. Understanding the impact factors of UGS on human health behaviors is a research priority and frontier, concerning both qualitative and quantitative methods. Different physical health effects and psychological benefits are linked to different characteristics of UGS, which is significant (39, 42, 52, 53). There are two typical kinds of features regarding UGS. One is the particular characteristics and attributes of UGS, referring to size, area, aesthetic characteristics, landscape quality, functional infrastructure, etc. Specifically, the certain functional infrastructure (i.e., trees, lawns, flowerbeds, and play and outdoor fitness equipment) and aesthetic factors of UGS can promote adolescents' health exercise (54, 55). Also, residents prefer to use parks with distinctive designs (45). Another one is the features related to the external environment, such as distribution, distance, and accessibility of UGS. More specifically, the accessibility and distance of UGS can affect human behavior, such as the levels of walking (40). Most studies proved that both have a positive and significant relationship with HB (22). Hence, well-planned, well-designed, and better features of UGS may lead to increased usage and physical activity (56).

Last but not least, due to the many benefits of green space to human health, when green space becomes a special or even a scarce resource, it will inevitably involve environmental and social inequity. For instance, Gordon-Larsen et al. (43) believe that inequality in the availability of UGS for lower socioeconomic status groups may lead to overweight. Recently, some studies focus on the fair access and use of UGS concerning health behaviors and outcomes, especially for different age groups (youth, adults, and old adults) (15, 38, 57), different gender groups (18, 58), people of different race/ethnicity (e.g., white, black, and Hispanic) and income groups (59). For socially disadvantaged groups, whether UGS is provided fairly or not is a vital issue in the field of social and environmental justice (60). Most scholars point out that it is necessary to rationally plan the spatial distribution of UGS, and solve the problem of inequity by supplementing enough UGS and shortening the accessible distance. However, this is a contradictory issue that creating new UGS also increases housing costs and property values (5).

## Conclusion

The results show the growing number of published articles from 2002 to 2021, with the number of published papers increasing from 1 in 2002 to 131 in 2021. The publication source with the highest circulation is the *International Journal of Environmental Research and Public Health*. Most important articles were published in journals dealing with the environment, and public health studies. The UGS and HB studies cover a total of 93 disciplines and 73 research directions. Public Environmental & Occupational Health and Environmental Sciences are the two most important disciplines. Research is more concentrated in the natural sciences. The United States had the highest number of publications, and Australia had the highest betweenness centrality value. The most productive institutions are the University of Hong Kong and the University of Exeter. A total of 11 clusters were extracted by literature co-citation analysis. The largest cluster was “human health”. The keyword co-occurrence analysis obtained 19 words with the highest co-occurrence frequency and 20 words with the highest betweenness centrality value. Plus, “physical activity” is the most important keyword. Furthermore, the keywords mainly involve three research objects (different scales of UGS; different health behaviors and outcomes; the impact factors). In the end, there are three research frontier trends: (1) the relationship between UGS and HB; (2) the influence factors; (3) the study of environmental justice and social equity.

## Data availability statement

The original contributions presented in the study are included in the article/supplementary material, further inquiries can be directed to the corresponding author/s.

## Author contributions

SZ conceived and designed the study, conducted the systematic review and analyzed the data, and as well as wrote the manuscript. ZC contributed significantly to analysis and manuscript preparation. YO visualized the data and made the tables. SZ and XL reviewed and refined the paper. All authors contributed to the article and approved the submitted version.

## Funding

This work was supported by the National Natural Science Foundation of China (NSFC) (Grant No. 52008345) and the Natural Science Foundation of Sichuan Province (Grant No. 2022NSFSC1108).

## Conflict of interest

The authors declare that the research was conducted in the absence of any commercial or financial relationships that could be construed as a potential conflict of interest.

## Publisher's note

All claims expressed in this article are solely those of the authors and do not necessarily represent those of their affiliated organizations, or those of the publisher, the editors and the reviewers. Any product that may be evaluated in this article, or claim that may be made by its manufacturer, is not guaranteed or endorsed by the publisher.
